# Electrochemical Biosensors 3D Printed by Fused Deposition Modeling: Actualities, Trends, and Challenges

**DOI:** 10.3390/bios15010057

**Published:** 2025-01-17

**Authors:** Luiz Ricardo Guterres Silva, Carlos Eduardo Costa Lopes, Auro Atsushi Tanaka, Luiza Maria Ferreira Dantas, Iranaldo Santos Silva, Jéssica Santos Stefano

**Affiliations:** 1 Graduate Program in Chemistry, Federal University of Maranhão, São Luís 65080-805, MA, Brazil; luizricardogus@gmail.com (L.R.G.S.); carlosedu707@hotmail.com (C.E.C.L.); tanaka@ufma.br (A.A.T.); luiza.dantas@ufma.br (L.M.F.D.); iranaldo.ss@ufma.br (I.S.S.); 2 Department of Chemistry, Federal University of Maranhão, São Luís 65080-805, MA, Brazil; 3 Department of Chemical Technology, Federal University of Maranhão, São Luís 65080-805, MA, Brazil

**Keywords:** FDM 3D printing, electrochemical biosensors, selective detection, virus, biomarkers, glucose

## Abstract

The technology of 3D printing, particularly fused deposition modeling (FDM) 3D printing, has revolutionized the development of electrochemical biosensors, offering a versatile and cost-effective approach for clinical applications. This review explores the integration of FDM in fabricating biosensing platforms tailored for clinical diagnostics, emphasizing its role in detecting various biomarkers and viral pathogens. Advances in 3D printing materials, especially the emergence of bespoke conductive filaments, have allowed the production of highly customizable and efficient biosensors. A detailed discussion focuses on the design and application of these biosensors for viral detection, highlighting their potential to improve diagnostic accuracy. Furthermore, the review addresses current trends, including the push towards miniaturization and multianalyte detection, alongside challenges such as material optimization and regulatory hurdles. By providing a comprehensive overview, this work underscores the transformative impact of 3D-printed electrochemical biosensors in clinical diagnostics while also identifying critical areas for future research and development.

## 1. Introduction

### 1.1. Electrochemical Biosensors: Principles and Applications

**Figure 1:**
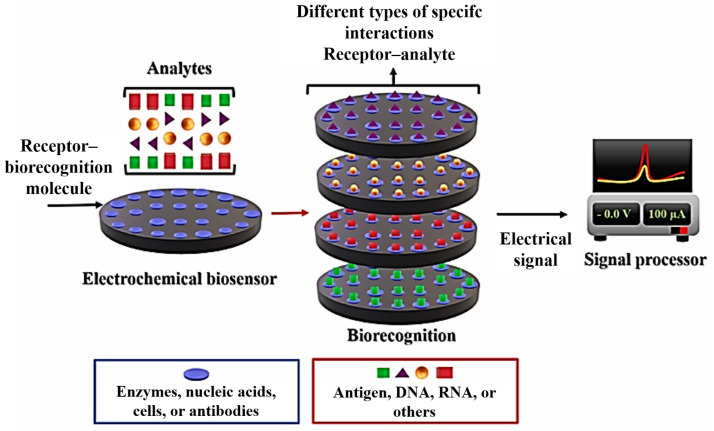
Scheme representative of a biosensor with the electrochemical transducer. Copyright (2023), Elsevier [7].

Electrochemical biosensors are highly sensitive analytical tools relying on the interaction between a specific biological agent and the target analyte [1,2,3,4]. These biological agents, including enzymes, antibodies, proteins, nucleic acids, cells, or receptors, provide exceptional selectivity, enabling precise detection of target substances. The operating principle of these devices involves the conversion of biochemical signals from the interaction between the analyte and the biological agent into measurable electrical responses, thereby providing a quantitative readout of the analyte concentration [3,4,5,6]. Figure 1 illustrates a general schematic of the action mechanism of an electrochemical biosensor.

The choice of the biological agent is crucial, as each type of biomolecule reacts specifically with the target substance, generating electrical signals proportional to its concentration or inducing changes in the electrochemical system that can be monitored and correlated with the analyte concentration [3,4]. The biological recognition system, therefore, plays a key role in interacting with the analyte in a highly selective and precise manner. The ability of electrochemical biosensors to detect and quantify a wide range of molecules, including food contaminants, bacteria, viruses, biomarkers, and even specific DNA sequences, makes these devices highly promising for clinical and diagnostic applications [3,8,9,10,11]. In these fields, the sensitivity and reliability of measurements are essential for rapid and accurate diagnostics [3].

Furthermore, advancements in manufacturing technologies, including photolithography, inkjet printing, silk screen printing, and additive manufacturing, have significantly contributed to the development of high-quality electrochemical biosensors. Each of these techniques has unique strengths. Photolithography excels in producing high-resolution features but requires cleanroom environments and is costly [12,13,14]. Inkjet printing offers flexibility and cost-effectiveness, enabling the deposition of conductive inks, but is often limited to two-dimensional or slightly layered designs [15,16]. Silk screen printing stands out for its scalability and ability to create robust conductive layers, although it relies on predefined templates that can limit design versatility [17,18,19,20].

In this context, 3D printing, particularly through additive manufacturing techniques such as FDM, provides unique advantages. Its ability to fabricate complex, fully three-dimensional architectures enables the integration of structures such as microfluidic channels, multianalyte detection systems, and complex geometric designs that improve the applicability of the sensors. Unlike other methods, 3D printing combines customization, rapid prototyping, and material versatility, allowing for the creation of highly tailored and multifunctional devices. These attributes position 3D printing as a key technology in advancing electrochemical biosensor development, bridging the gap between laboratory innovation and scalable, real-world applications in healthcare and diagnostics [21,22].

In this aspect, the present review explores the use of 3D-printed electrochemical biosensors, specifically emphasizing their development, applications, potentialities, and perspectives. The role of 3D-printed biosensors is addressed here, particularly focusing on virus detection, a highly relevant topic given the global demand for rapid and precise diagnostics during the COVID-19 pandemic. Moreover, this review explores the latest advancements in the construction of conductive filaments, discussing their tailored properties and contributions to enhancing the performance of biosensors. By bridging the gap between novel filament development and practical biosensor applications, we not only address underexplored challenges but expand the discussions to include the feasibility of commercializing these platforms. This comprehensive approach provides a distinctive perspective, offering valuable insights into the current scenario and future directions of 3D-printed electrochemical biosensors.

### 1.2. Three-Dimensional Printing

There are various fields within 3D printing, offering innovative solutions that meet contemporary needs. In society, this technology enables the customization of products, from tailored prosthetics to educational models and personalized devices, facilitating access to solutions adapted to individual needs [21,23,24]. In industry, 3D printing optimizes manufacturing processes, reducing costs and production times, while also enabling rapid prototyping, which accelerates the development of new products. Sectors such as healthcare, automotive, aerospace, and architecture have already benefited from the versatility and efficiency of this technology, which drive innovation and sustainability [21,22].

An excellent example of 3D printing’s versatility was seen during the SARS-CoV-2 pandemic, where it played a crucial role in manufacturing personal protective equipment (e.g., face shields, masks) and sample collection devices (e.g., swabs) [25,26,27]. Beyond healthcare, 3D printing has made significant contributions to electrochemical applications, with notable advancements in the production of energy storage devices, CO_2_ capture, ammonia production, and sensors for environmental monitoring [28,29,30,31,32]. These examples underscore the wide-ranging potential of 3D printing technology and highlight its distinctive and innovative contributions, especially in the development of electrochemical biosensors.

Among the various 3D printing techniques, FDM stands out primarily for its low cost, ease of operation, and rapid implementation, making it an accessible and efficient option for a wide range of industrial and commercial applications. Furthermore, the materials used in this 3D printing method are inexpensive, widely available on the market, have different types and compositions, and are easy to store, robust, and nontoxic for contact and handling.

In this sense, it is possible to infer that 3D printing is a transformative technology addressing diverse needs in society and industry, enabling customized solutions and efficient manufacturing across sectors such as healthcare, aerospace, and architecture. Its versatility was evident during the SARS-CoV-2 pandemic, where it facilitated the production of essential equipment and advanced electrochemical applications. The accessibility and efficiency of FDM further highlight its potential for widespread use and innovation.

### 1.3. Fused Deposition Modeling

FDM 3D printing is one of the most common techniques, widely used to create three-dimensional objects from digital models. The process begins with the design of a virtual prototype, which is generated using specialized software. These programs enable the creation of complex three-dimensional models, adjusting dimensions and applying specific details according to the project’s requirements [21,23,33].

**Figure 2:**
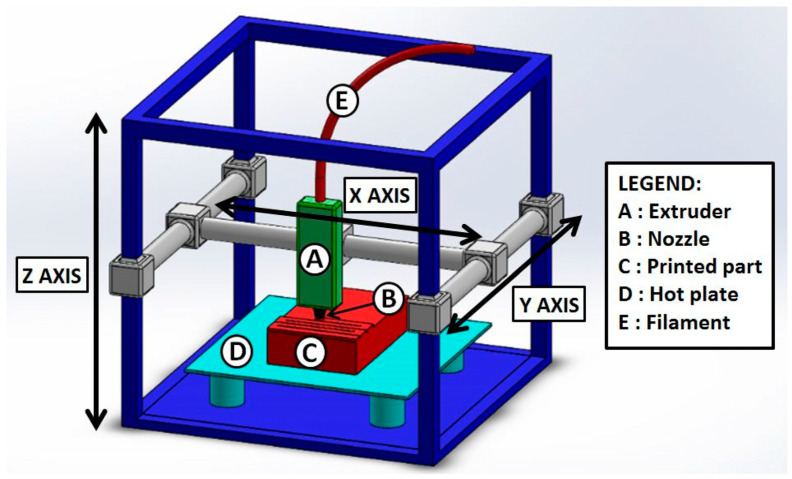
Schematic representation of a typical fused deposition modeling setup. Copyright (2019), MDPI [34].

In the FDM process, a thermoplastic filament is heated to a molten state and then extruded layer by layer to build the object. As each layer is deposited, it solidifies, forming a three-dimensional structure [22,33,34]. This method is widely adopted because of its simplicity, low operational cost, and ability to use a wide variety of materials, allowing for the production of objects with customized and specific characteristics for different applications. Figure 2 illustrates the manufacturing mechanism of objects using FDM 3D printing.

FDM 3D printing is a widely adopted technique for creating three-dimensional objects by extruding molten thermoplastic layer by layer. It stands out for its simplicity and versatility in using various materials to produce customized structures. This approach enables the fabrication of complex designs tailored to specific applications across diverse fields.

### 1.4. Three-Dimensionally Printed Electrochemical Sensors

In the development of electrochemical devices, FDM 3D printing offers numerous advantages. This technique allows the creation of complex and miniaturized geometries, tailored to the desired objective [21,22,33,35]. Additionally, automated large-scale production is a significant advantage of this approach. The application of 3D printing in the fabrication of electrochemical devices, such as sensors, has been made possible by the advent of conductive filaments, which are essentially a mixture of thermoplastic polymers and conductive materials [21,22]. This innovation has rapidly driven the expansion of 3D-printed electrochemical sensors, with the FDM model being the most widely used technique [21,22].

Electrochemical sensors 3D printed using the FDM technique are extensively documented in the literature, featuring a wide range of geometries, sizes, and applications [21,22,23]. These devices have been employed in the detection of pharmaceuticals, biomarkers, pesticides, metals, and other classes of compounds of interest. In recent years, 3D-printed electrochemical biosensors have gained prominence, particularly during the SARS-CoV-2 pandemic, which intensified their development [36,37,38,39,40]. These biosensors stand out because of their miniaturization capabilities, versatile design—which facilitates their use in point-of-care systems—and possibility of being disposable or recyclable [41,42,43].

Polylactic acid (PLA) is the most commonly used thermoplastic polymer in the production of conductive filaments for (bio)sensors [21,22]. It is a biodegradable, nontoxic, and highly sustainable material, derived from the fermentation of plant sources such as corn and cassava, which are natural and renewable. PLA is cost-effective, widely available, and has a relatively low printing temperature (180 to 220 °C), making it suitable for use in low-cost FDM 3D printers [34].

Commercial conductive filaments, such as Black Magic^®^ (containing a mixture of PLA and graphene) and Proto-Pasta^®^ (containing PLA and carbon black), have enabled the fabrication of several sensors and biosensors through 3D printing. These filaments rely on carbon-based nanomaterials such as graphene and carbon black to provide conductivity, with loadings of 8 wt.% graphene and 21 wt.% carbon black, respectively. While these materials demonstrate sufficient conductivity for certain applications, they were not specifically designed or optimized for sensor fabrication. Additionally, the presence of impurities, particularly trace metals, in commercial filaments can interfere with the electrochemical response and hinder their suitability for biosensing applications.

The development of bespoke conductive filaments has addressed many of these limitations by allowing the incorporation of conductive materials in higher and more precise proportions tailored specifically for sensor applications. By using materials such as graphite, carbon nanotubes, and advanced carbon-based nanomaterials, researchers have achieved significant enhancements in sensor performance, including sensitivities, lower detection limits, and increased reproducibility [44]. Moreover, the possibility to customize filament compositions often eliminates the need for laborious postprocessing steps such as surface treatments. This approach not only enhances the quality and functionality of 3D-printed (bio)sensors but expands the possibilities for designing devices with superior electrochemical properties.

In this presented context, FDM 3D printing has revolutionized the fabrication of electrochemical devices, enabling the creation of complex, miniaturized geometries and facilitating large-scale automated production. The development of conductive filaments, particularly those based on carbon-based nanomaterials like graphene and carbon black, has expanded the capabilities of 3D-printed sensors and biosensors, especially during the SARS-CoV-2 pandemic. Advances in bespoke conductive filaments have further improved sensor performance, offering enhanced sensitivity, reproducibility, and efficient manufacturing processes tailored to specific applications.

## 2. Three-Dimensionally Printed Electrochemical Biosensors

The first 3D-printed electrochemical biosensor using the FDM method was reported in 2019, to the best of our knowledge, utilizing a filament containing graphene as the conductive material [45]. Since then, approximately twenty-three scientific articles have been published, with a primary focus on biosensors for glucose, hydrogen peroxide, and the detection of the SARS-CoV-2 virus. Table 1 summarizes the 3D-printed biosensors described in the literature along with their key components and target analytes of interest.

### 2.1. Glucose 3D-Printed Biosensor

The development of biosensors for glucose quantification is crucial in public health because of the growing prevalence of diabetes, one of the leading causes of morbidity and mortality global. Effective and continuous blood glucose monitoring is essential for preventing severe complications such as cardiovascular diseases, neuropathies, and renal failure. Biosensors offer a fast, precise, and accessible solution, facilitating glycemic control, promoting patients’ quality of life, and reducing costs associated with the treatment and management of diabetes on a large scale. In this context, 3D-printed electrochemical biosensors have been widely explored and reported because of the ongoing need for glucose sensors.

The first 3D-printed biosensor reported for glucose detection was presented by Katseli et al. (2019) [45]. In their study, the authors used a dual-extruder 3D printer to produce an electrochemical cell integrated with three electrodes (reference, counter, and working). The commercially available conductive filament used for electrode production was based on carbon and PLA, and later, the working electrode was modified with glucose oxidase by immersing it in a solution containing a mixture of glucose oxidase and Nafion. The key point highlighted by the authors was the miniaturization of the system and the ability to produce it in a single step using the dual-extruder printer, making it ideal for point-of-care applications.

**Figure 3:**
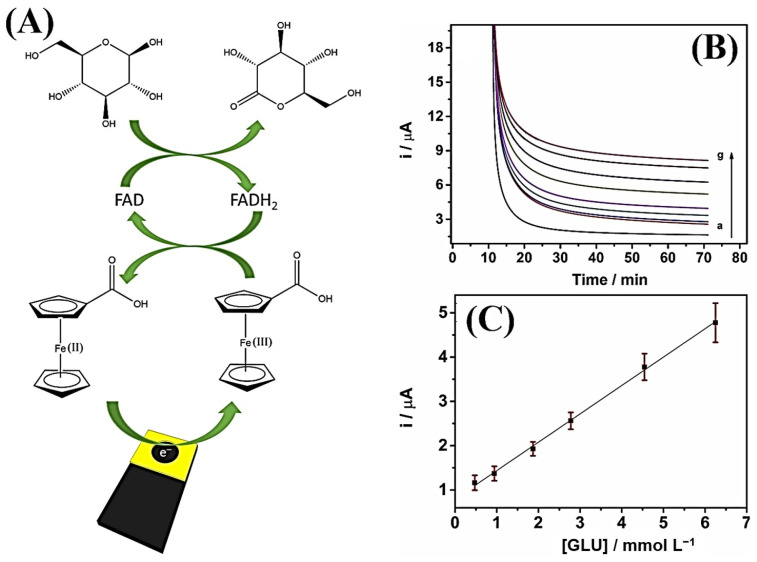
(**A**) Representation of the GOx biosensor in two steps to convert glucose into glucolactone. (**B**) Amperometric curves obtained in the presence of glucose (a: 0; b: 0.5; c: 1.0; d: 1.8; e: 2.8; f: 4.6 and g: 6.3 mmol L^−1^). (**C**) Linear regression obtained from limit current versus glucose concentration. Copyright (2020), ELSEVIER [48].

Cardoso et al. (2020) [48] developed a 3D-printed electrode using commercial conductive filaments based on graphene and PLA and subsequently anchored glucose oxidase onto the electrode surface by crosslinking with glutaraldehyde for glucose determination in blood plasma. A schematic of the designed electrode, the mechanism of action for glucose detection, and the results of the analyses can be seen in Figure 3. The sensor was printed in a rectangular shape with the analysis area delimited by vinyl adhesive. Glucose oxidase was immobilized on the surface using a simple drop-casting method, and glucose analysis was performed through amperometry. The detection in fortified blood plasma samples showed recoveries close to 100%. The 3D-printed device demonstrated good applicability, rapid analysis, and miniaturization—essential characteristics for the development of new bioanalytical devices.

Wang and Pumera (2021) [52] fabricated a 3D-printed nanocarbon electrode, and the biosensor was developed through the immobilization of glucose oxidase by covalently binding the enzyme to the electrode surface. The produced electrode was used for multiplexed biodetection of glucose and hydrogen peroxide. The electrode was initially chemically treated with DMF to remove PLA from the surface; this was followed by the addition of the EDC:NHS compound to the sensor surface, serving as a crosslink for glucose oxidase anchoring. According to the authors, the construction of the sensor is highly practical and can be applied on a large scale, with real-world potential for glucose and hydrogen peroxide monitoring and control.

Domingo-Roca et al. (2022) [54] reported a study where they developed a complete electrochemical system consisting of three integrated electrodes for microvolume analysis (100 µL). The electrodes were fabricated from commercially available conductive filaments based on carbon black and PLA. For modification of the working electrode, a functionalization protocol was employed based on the enzymatic entrapment of glucose oxidase in a gelatin hydrogel crosslinked on the electrode surface. Continuing in the same line of fully integrated systems, Calabria et al. (2023) [55] developed a unique and intriguing work on a miniaturized and portable electrochemiluminescence-based biosensor, 3D-printed for glucose detection in artificial human serum and glucose saline solutions. The biosensor was obtained by coupling the luminol/H_2_O_2_ system with glucose oxidase in an agarose matrix. Glucose detection was performed using electrical signal and light emission, thus combining two reliable and easy-to-operate techniques.

When comparing the various studies on glucose determination, it is evident that despite differences in device architecture, filament selection, and other factors, the use of glucose oxidase remains central. The versatility of 3D printing is highlighted, enabling the fabrication of both simple, miniaturized systems with integrated three-electrode configurations for drop analysis and more complex setups such as integrated electrochemiluminescence systems. Regarding analytical characteristics, while some studies did not report the LOD, those that did presented similar values in the micromolar range. Additionally, the reported stability and reproducibility across different designs demonstrate the robustness and reliability of these biosensors. The advancement of these devices, combined with 3D printing, opens new perspectives for the creation of customized, accessible, and effective biosensors capable of meeting the growing demand for practical solutions in diabetes management and improving patients’ quality of life.

### 2.2. SARS-CoV-2 3D-Printed Biosensors

The need for rapid and reliable analytical tests for virus detection became evident during the COVID-19 pandemic, highlighting the importance of early and accessible diagnostics to contain the spread of diseases. In this context, 3D-printed electrochemical biosensors have emerged as a promising alternative. This technology offers a versatile and efficient solution, particularly for point-of-care applications, in response to global health crises. Therefore, in this section, we focus on 3D-printed biosensors for the detection of different types of viruses. Initially, we discuss 3D-printed biosensors for detecting various biomarkers of the SARS-CoV-2 virus.

**Figure 4:**
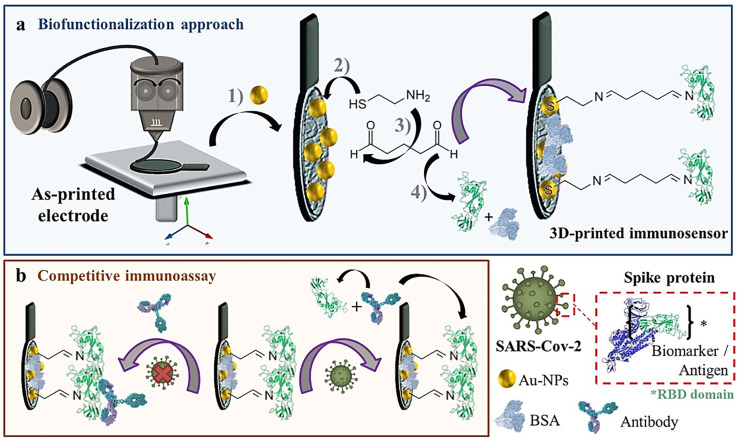
(**a**) Illustration of the 3D-printed electrochemical COVID-19 immunosensor fabrication steps, (1) in situ incorporation of gold nanoparticles, (2) anchoring of a thiolated moiety, (3) anchoring of the cross-linker, and (4) immobilization of biomarker and bovine serum albumin blocking. (**b**) Indirect competitive assay carried out for detecting the COVID-19 recombinant protein (antigen), the one against the SARS-CoV-2 virus. * COVID-19 Spike Protein RBD Domain Coronavirus. Copyright (2021), ELSEVIER [39].

Professor Pumera’s research group explored the application of 3D-printed biosensors for SARS-CoV-2 detection. Muñoz and Pumera (2021) [39] developed the first 3D-printed biosensor to detect the virus using commercial graphene and PLA filaments. The device was constructed through bottom-up biofunctionalization, covalently anchoring the recombinant virus protein onto the working electrode surface. Detection was carried out by electrochemical impedance spectroscopy, monitoring changes at the electrode/electrolyte interface during a competitive assay with monoclonal antibody, demonstrating high sensitivity and potential for point-of-care applications. Figure 4 shows a schematic of the biosensor’s construction and its interaction mechanism with the target analyte. Another significant work by the group was that of Crevillen et al. (2022) [36], who developed a lab-on-a-chip system, combining microfluidics with a fully 3D-printed electrochemical cell. The device, modified with a ssDNA probe targeting the N gene of SARS-CoV-2, detects viral RNA through the electro-oxidation of adenines in the ssDNA, with RNA–target hybridization causing the desorption of ssDNA and the reduction of the electrochemical signal. This device stands out for its high sensitivity, selectivity, and rapid response, with great potential for use as a rapid, noninvasive test.

**Figure 5:**
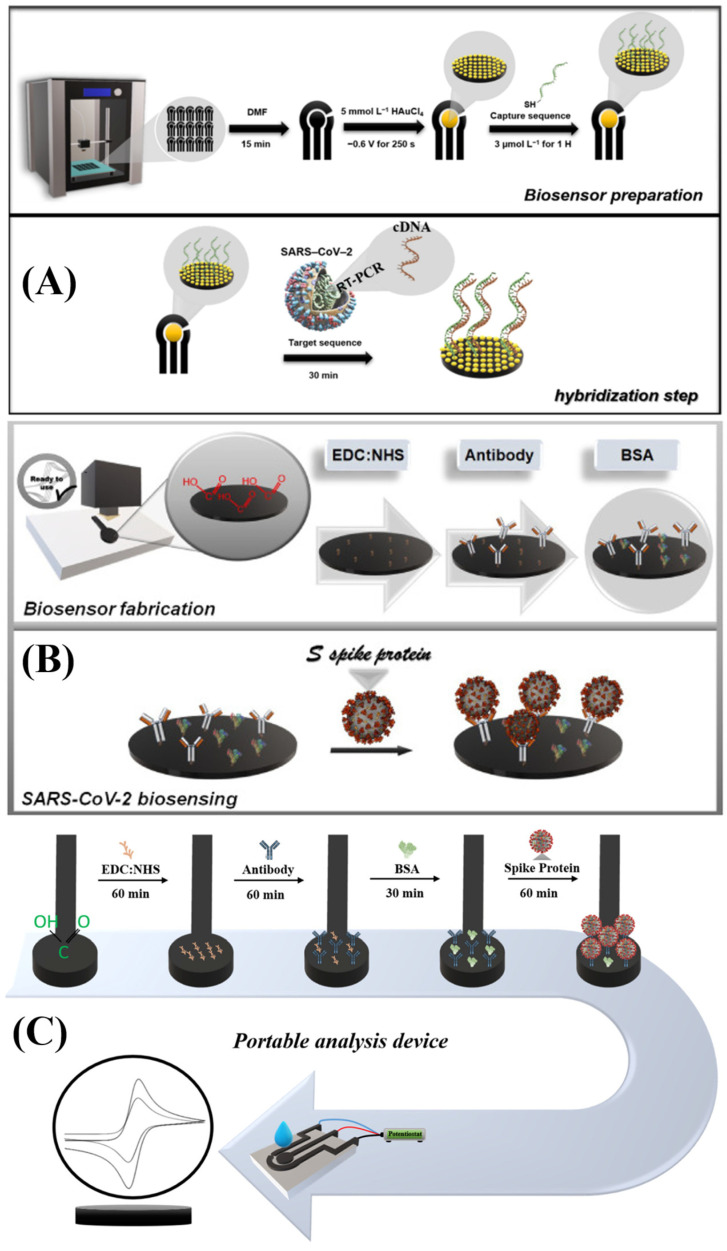
(**A**) Schematic illustration of the production of the 3D-printed electrochemical genosensor and hybridization step. (**B**) Representative scheme of the involved steps in the fabrication of the 3D-printed electrochemical immunosensor. (**C**) Scheme of all steps involved in the construction of the electrochemical immunosensor and conception of the real design of the electrochemical sensor. (**A**) Copyright (2022), MDPI [59]. (**B**) Copyright (2022), ELSEVIER [58]. (**C**) Copyright (2023), ELSEVIER [38].

The research group led by Professor Janegitz has explored the potential of 3D-printed biosensors for the detection of different biomarkers of SARS-CoV-2. Silva et al. (2022) [59] developed a genosensor based on graphene and PLA filaments, inspired by disposable electrodes, for 50 µL analyses, using gold nanoparticles to detect the virus through hybridization of cDNA with monitoring by a redox probe. Stefano et al. (2022) [58], on the other hand, created conductive graphite and PLA lab-made filaments to fabricate an immunosensor for the spike S1 protein, enabling rapid and selective analyses with only 150 µL, ideal for point-of-care applications. In a similar vein, Silva et al. (2023) [40] employed carbon black and PLA-based lab-made filaments to create an immunosensor for the detection of the SARS-CoV-2 spike S1 protein. The sensor was designed to be miniaturized for analysis in 50 µL droplets. Figure 5 illustrates the production methods for the three works developed.

**Figure 6:**
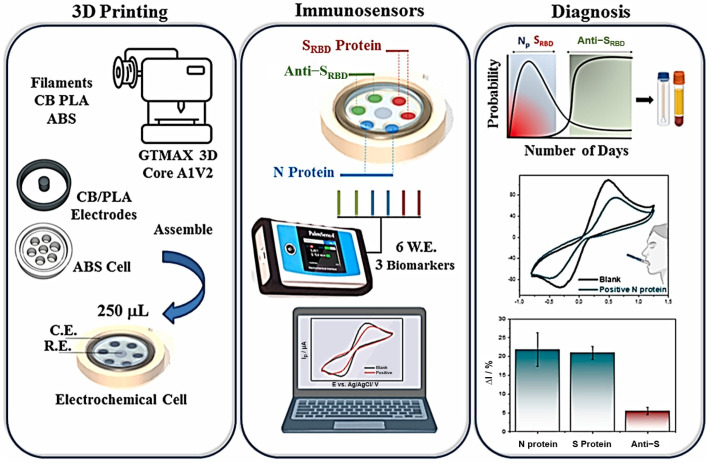
Illustrative representation of the 3D-printed cell based on six electrodes and the corresponding electrochemical configuration for multiplexed detection. Copyright (2023), ELSEVIER [40].

Morawski et al. (2023) [40] developed a 3D-printed electrochemical biodevice consisting of six working electrodes for the simultaneous detection of three SARS-CoV-2 virus biomarkers. The sensors were fabricated using a commercial carbon black and PLA-based filament, and analyses were performed with a 250 µL aliquot. Figure 6 illustrates the steps involved in the process. The biomarkers detected were the N-protein, the SRBD-protein, and anti-SRBD. The sensor demonstrated good selectivity for analyzing these biomarkers and was also effective in analyzing fortified human serum. This device highlights the potential of 3D printing, combined with conductive filaments, for producing complex and multiplex devices.

The production of conductive filaments in the laboratory has played a crucial role in the development of highly efficient electrochemical biosensors, as discussed earlier. The manufacturing methods employed in previous works relied on mixing thermoplastic polymers, such as PLA, with carbon-based compounds in appropriate solvents, resulting in homogeneous and conductive filaments. This approach represents a viable and cost-effective alternative to commercial conductive filaments, allowing for greater control over the composition and properties of the material. Furthermore, laboratory-produced filaments eliminate the need for additional surface treatments for biosensor fabrication, simplifying the production process. This formulation flexibility enables the optimization of filaments for better analytical response, paving the way for the development of increasingly sensitive biosensors tailored to specific applications.

Three-dimensionally printed electrochemical biosensors have proven to be an innovative and effective solution for detecting SARS-CoV-2 biomarkers, particularly in the context of global health crises such as the COVID-19 pandemic. The use of commercial or custom-made laboratory conductive filaments has enabled the production of high-performance sensors, including genosensors and immunosensors, capable of performing analyses in microvolumes. Additionally, the demonstration of 3D printing technology’s ability to produce multiplexed devices capable of detecting multiple biomarkers in a single sample highlights its potential for diagnostics suitable for point-of-care use. These technological advancements emphasize the importance of 3D printing in producing accessible and accurate sensors capable of meeting the growing demand for rapid and reliable solutions during public health emergencies.

### 2.3. Three-Dimensionally Printed Electrochemical Biosensors for Different Targets

The search for new detection targets in biosensors is crucial to enhance diagnostic capabilities and support public health. Detecting analytes such as cholesterol, hydrogen peroxide, viruses (such as yellow fever and monkeypox), tumor biomarkers, tryptophan enantiomers, and substances associated with Parkinson’s disease is essential for early diagnosis, monitoring, and treatment of various health conditions. Advancements in these areas can significantly contribute for disease prevention and management, as well as improving responses to infectious outbreaks and managing chronic and neurodegenerative diseases. This section discusses works reporting 3D-printed biosensors for different clinically relevant analytes.

**Figure 7:**
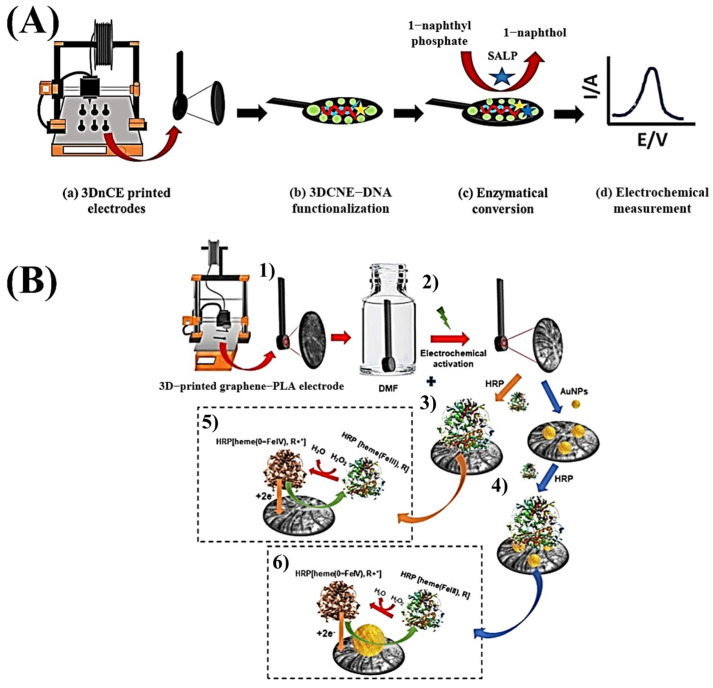
(**A**) Schematic representation of the analytical protocol for production of biosensor. (**B**) Scheme of 3D graphene-PLA biosensor fabrication, (1) 3D printing of the electrode; (2) activation in DMF and via electrochemical methods; (3) modification of the 3D-printed electrode with the HRP enzyme; (4) functionalization of the electrode with gold nanoparticles, followed by immobilization of the HRP enzyme, (5) and (6) are corresponding mechanisms of H_2_O_2_ detection. (**A**) Copyright (2023), ELSEVIER [60]. (**B**) Copyright (2020), ELSEVIER [47].

The research group led by Professor Pumera has explored various approaches in the fabrication of biosensors using commercial conductive filaments. Manzanares-Palenzuela et al. (2019) [46] developed 3D-printed graphene/PLA biosensors for the detection of 1-naphthol, utilizing alkaline phosphatase to convert 1-naphthyl phosphate into 1-naphthol, which was then detected through electrochemical oxidation. Jyoti et al. (2023) [60] created 3D-printed nanocarbon electrodes (3DnCes) for DNA detection, using 1-naphthol generated by alkaline phosphatase as the analytical signal. Marzo et al. (2020) [47] developed a graphene/PLA biosensor by immobilizing peroxidase and gold nanoparticles to detect hydrogen peroxide via electron transfer, with biomedical applications. Figure 7 illustrates the manufacturing methods of the biosensors presented by Jyoti et al. (2023) and Marzo et al. (2020).

Muñoz et al. (2021) [51] investigated 3D-nCEs biofunctionalized with L-amino acid oxidase for chiral analysis, using impedimetric monitoring to detect changes in the electrode interface caused by the interaction with H_2_O_2_, allowing for ultrasensitive discrimination of enantiomers. Wang et al. (2021) [53] developed a 3D-printed chiral sensor functionalized with a covalent organic framework (COF) and bovine serum albumin as the chiral surface. Using tryptophan as a model, the Fe_3_O_4_@COF@BSA/3DE sensor demonstrated high selectivity for L-Trp over D-Trp, enabling quantification in racemic mixtures. This approach expands the possibilities for on-demand fabrication of modified electrodes, with applications in enantiomer recognition.

**Figure 8:**
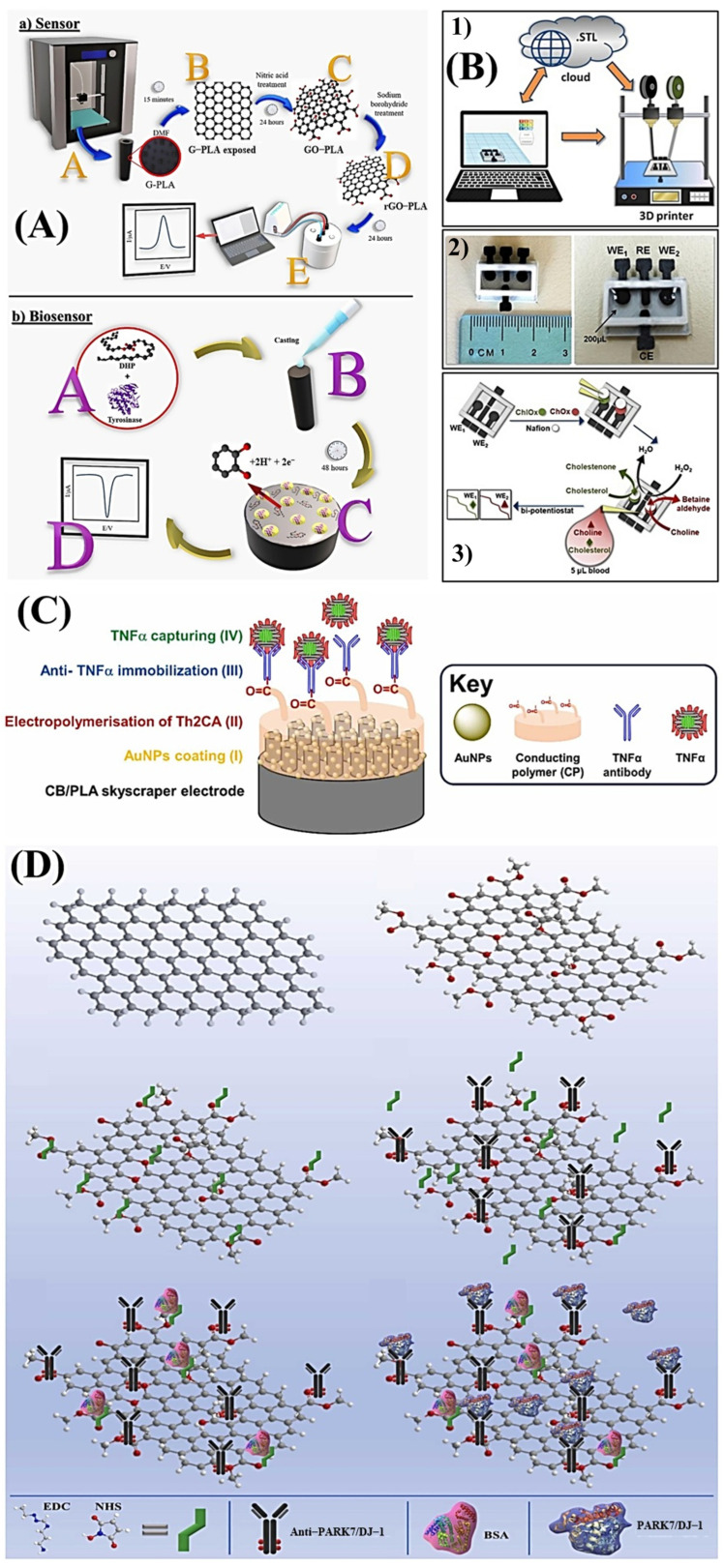
(**A**) Scheme of the procedure for obtaining the G-PLA 3D-printed working, counter, and reference electrodes and biosensor production. (**B**) (1) Fabrication procedure of the 3D printed e-transferable microchip, (2) photographs of the microchip, and (3) the construction process of the biosensors. (**C**) Schematic representation for the stepwise fabrication of the skyscraper immunosensor. (**D**) 3D-printed immunosensor based on graphene immobilization scheme. (**A**) Copyright (2020), ELSEVIER [49]. (**B**) Copyright (2021), ELSEVIER [50]. (**C**) Copyright (2024), ELSEVIER [56]. (**D**) Copyright (2023), ELSEVIER [61].

Silva et al. (2020) [49] developed a compact electrochemical biosensor printed in 3D using commercial graphene and PLA filaments, creating a rGO-PLA electrode for catechol determination. The working electrode was modified with a layer of dihexadecyl phosphate enzyme. Koukouviti and Kokkinos (2021) [50] created a four-electrode biochip printed in 3D with carbon black and PLA filaments, capable of simultaneously detecting cholesterol and choline in a drop of blood, offering detection limits below cutoff levels for coronary syndromes. Hussain et al. (2024) [56] designed a robust sensor using 3D printing to create electrode structures resembling skyscrapers, immobilizing anti-TNFα and gold nanoparticles; this sensor was capable of monitoring levels of tumor necrosis factor-alpha (TNFα) in fecal pellets. Kalinke et al. (2023) [61] developed a 3D-printed electrode array from commercial graphene and PLA conductive filaments for detecting PARK7/DJ-1 protein in serum and cerebrospinal fluid samples, using chemical and electrochemical activation to enhance antibody immobilization, showing good repeatability and reproducibility for diagnosing Parkinson’s disease. These advancements demonstrate the potential of 3D printing in the creation of cost-effective, sensitive, and adaptable sensors and biochips. Figure 8 illustrates the construction steps of the biosensors discussed in each study.

**Figure 9:**
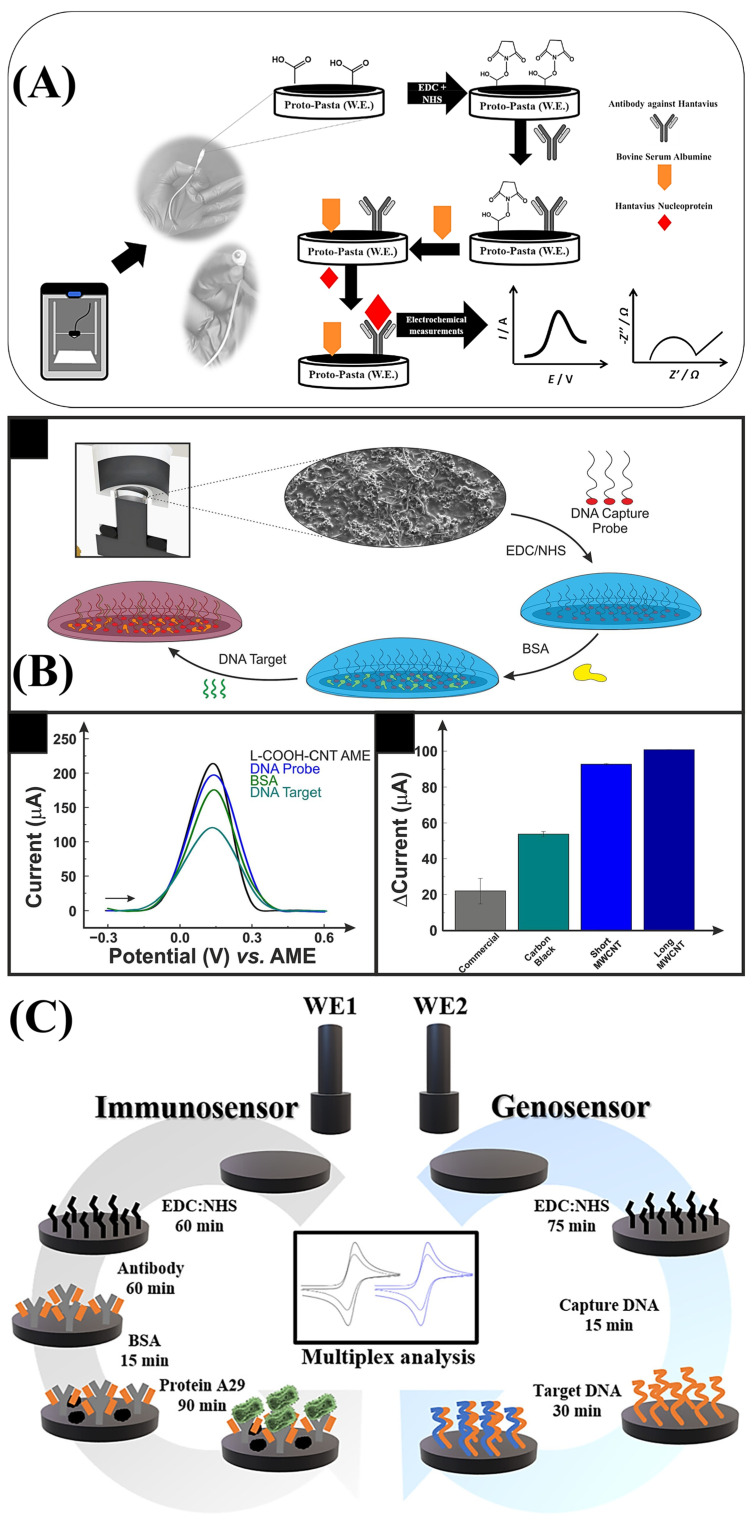
(**A**) Immunosensor step by step buildup. (**B**) Schematic representation of the genosensor’s production by drop-casting. (**C**) Illustrative diagram of the biosensor’s preparation steps. (1) Copyright (2020), ELSEVIER [57]. (2) Copyright (2023), ELSEVIER [43]. (3) Copyright (2024), ELSEVIER [37].

The development of 3D-printed electrochemical biosensors for virus detection beyond SARS-CoV-2 has also been investigated. Martins et al. (2021) [57] presented the first 3D-printed electrochemical immunosensor using commercial carbon black and PLA filament to detect the nucleoprotein of Hantavirus Araucaria. The recognition biomolecule was anchored on the filament surface through EDC/NHS coupling, demonstrating the device’s potential for the quantitative detection of the nucleoprotein in diluted human serum samples. The use of PLA-based conductive filaments with carbon black, featuring carboxyl groups that enable the covalent anchoring of biomolecules, provides a simple and efficient platform for immunosensors. Customized conductive filaments have also been employed in biosensors for the detection of other viruses. Kalinke et al. (2023) [43] utilized recycled PLA, carbon black, and carbon nanotubes (COOH-MWCNT) to fabricate a high-performance genosensor for detecting cDNA from the yellow fever virus in human serum samples. Silva et al. (2024) [37] developed a multiplexed platform based on two 3D-printed biosensors for detecting biomarkers of the monkeypox virus, including a genosensor for target DNA and an immunosensor for the A29 protein. The multiplex device represents a promising alternative for rapid and portable point-of-care detection. Figure 9 illustrates the construction steps of the biosensors discussed in each study.

The studies discussed in this section demonstrate the potential of these biosensors to detect analytes such as viruses, tumor biomarkers, and substances associated with neurodegenerative diseases, highlighting their application in public health environments, rapid diagnostics, and continuous monitoring. The development of new platforms and the ongoing evolution of the materials used are crucial for further expanding the application possibilities of these devices, offering innovative solutions to contemporary clinical challenges.

## 3. Conclusions: Trends and Challenges

FDM 3D printing has been established as a transformative technology in the development of electrochemical biosensors, offering versatility and efficiency with great affordability. A notable trend in this area is the extensive use of commercial conductive filaments, enabling the fabrication of highly sensitive, low-cost biosensors. Among the studies reviewed, 19 employed commercial filaments, evidencing their accessibility and reliability. However, the next scientific breakthrough in this area lies in the development of lab-made conductive filaments, which allow customization and optimization of printing. By integrating advanced materials such as carbon nanotubes, the performance of the electrochemical biosensors can be significantly improved. Although fewer studies have explored this approach, incorporating high-quality materials presents evident potential for innovation, allowing the fabrication of devices that meet specific needs.

The reproducibility of 3D-printed electrochemical biosensors is significantly influenced by printing parameters and the conditions of the materials employed. While different printer models can produce identical prototypes within their technical limitations, key factors such as printing orientation, material infill density, and printing temperatures play crucial roles in ensuring the consistent performance of the devices. These parameters directly affect the structural and electrochemical properties of the biosensors, highlighting the importance of optimizing and standardizing printing protocols. Moreover, the quality of the filament is critical: improper storage conditions, particularly exposure to humidity, can degrade the filament, decreasing its quality and compromising printing reliability and sensor performance. Studies have also shown that filament aging negatively impacts its conductivity, further emphasizing the need for proper material handling and storage practices to maintain reproducibility and functionality over time [62]. Addressing these practical challenges is essential for advancing the field and scaling the production of high-quality 3D-printed biosensors.

The development of lab-made conductive filaments has reached a significant level of maturity, with advancements such as the incorporation of plasticizers contributing significantly to the optimization of their mechanical properties and printability. This progress highlights their potential for real-world applications and commercialization. Solvent-based methods, while limited in scalability, are particularly valuable for laboratories with minimal infrastructure and for the development of new filament compositions during the research phase. However, when considering the large-scale production of conductive filaments, the adoption of efficient thermal mixing technologies stands out as the most viable pathway. Thermal methods enable the production of high-quality and reproducible filaments, aligning with industrial demands for large-scale manufacturing. Despite the higher initial investment, exploring cost-effective alternatives for thermal mixing equipment could make this approach more accessible and practical for continuous production.

To transform conductive filaments into commercial products, validation under industrial conditions is essential to ensure reproducibility and quality. Additionally, the ability to produce tailored filaments with optimized conductive properties, while minimizing surface treatment steps, further enhances their market potential. These tailored materials could bridge the gap between laboratory innovation and real-world applications, enabling the extensive fabrication of advanced 3D-printed biosensors. By addressing scalability challenges and refining production methods, lab-made conductive filaments could become high-quality tools for diagnostics and other applications.

Another growing trend is the miniaturization of devices, enabled by the increasing precision of 3D printing. This capability to create smaller and more compact biosensors directly enhances their portability and ease of use, critical factors for point-of-care diagnostic devices. In parallel, 3D printing is facilitating the development of multiplexed electrochemical biosensors capable of detecting multiple analytes on a single platform, thereby broadening their applications in rapid and reliable diagnostics. The creation of these platforms is relatively simple, often involving the design of devices that integrate multiple sensors. This approach can be achieved with simple, low-cost 3D printers, enabling users to develop functional prototypes efficiently.

Additionally, advances in 3D printer technology, such as the use of multinozzle (two or more) extruders, are simplifying the construction of multiplexed devices. These modern printers allow for rapid fabrication using different filaments simultaneously, enabling the creation of complex systems with multiple functionalities Such innovations reveal pathways for scalable and customizable biosensor platforms personalized to diverse diagnostic needs, as exemplified by viral monitoring applications during the COVID-19 pandemic. Despite these advancements, several challenges remain. The production of conductive filaments in the laboratory requires continuous improvement of formulations to enhance the quality and reproducibility of the materials. The incorporation of metal particles, such as gold or silver, to act as redox mediators represents a significant trend but still faces challenges related to the homogeneity and control of the electrochemical properties of these filaments. Furthermore, the fabrication of 3D-printed electrochemical biosensors must advance to make these devices more reliable and reproducible, particularly in terms of consistent performance over time and under varied conditions of use.

The transition of 3D-printed biosensors from laboratory research to real-world applications remains a significant hurdle, reflecting their current Technology Readiness Level of 3 to 5. While these devices demonstrate robustness, their use has largely been confined to simplified or artificial samples, highlighting the need for validation in complex, real-world scenarios. Key regulatory challenges include meeting rigorous standards for accuracy, durability, and patient safety, which vary significantly across countries and organizations. To overcome these hurdles, the first critical step is extensive testing in real patient samples, directly comparing performance against gold-standard diagnostic methods to establish equivalency or superiority.

Additionally, ensuring the commercial viability of these devices requires comprehensive studies on reproducibility, shelf-life, and storage conditions under diverse environmental factors. Developing robust protocols for manufacturing and quality control is equally crucial to guarantee consistent performance. Early collaboration with regulatory bodies can streamline the approval process by providing clarity on specific requirements for clinical validation and market entry. By addressing these aspects, 3D-printed biosensors can progress toward becoming reliable, scalable tools for healthcare and environmental diagnostics, ultimately bridging the gap between innovation and extensive application.

## Figures and Tables

**Table 1:** Three-dimensionally printed electrochemical biosensors reported in the literature and their characteristics and applications.

Biosensor	Conductive Material	Biorecognition Agent	Analyte	Sample	LOD	Ref
3D-printed biosensor	Graphene	Glucose oxidase	Glucose	-	-	[45]
3D-printed biosensor	Graphene	Alkaline phosphatase	1-naphthol	-	-	[46]
DMF-EC/AuNPs/ HRP	Graphene	Horseradish peroxidase	H_2_O_2_	Human serum	9.1 μmol L^−1^	[47]
G-PLA	Graphene	Glucose oxidase	Glucose	Blood plasma	15.0 μmol L^−1^	[48]
Tyr-DHP/rGOPLA	Graphene	Tyrosinase	Catechol	Water	0.26 μmol L^−1^	[49]
3D printed e-transferable microchip	CB	Cholesterol oxidase Choline oxidase	Cholesterol Choline	Blood	3.36 μmol L^−1^ 0.08 μmol L^−1^	[50]
L-AAO@3D-nCEs	Graphene	L-amino acid oxidase	L-alanine	-	1.0 μmol L^−1^	[51]
GOx/3DE	CB	Glucose oxidase	H_2_O_2_ Glucose	Apple cider	2.97 μmol L^−1^ 158.0 μmol L^−1^	[52]
Fe_3_O_4_@COF@BSA/ 3DE	Graphene	Magnetic covalent organic framework and bovine serum albumin	Tryptophan enantiomer	-	-	[53]
3D-printed platform	CB	Glucose oxidase	Glucose	-	-	[54]
3D-printed miniaturized ECL biosensor	CB	Glucose oxidase	Glucose	Artificial serum and glucose saline solutions	60.0 μmol L^−1^	[55]
SS electrode	CB	Anti-TNFα	Tumor necrosis factor alpha protein	Fecal pellets	44.6 pg mL^−1^	[56]
3D printed biosensor	CB	IgG2B antibody	Hantavirus Araucaria nucleoprotein	Synthetic saliva	22 μg mL^−1^	[57]
3D-printed immunosensor	Graphene	Recombinant protein	COVID-19 protein	Human serum	0.5 μg mL^−1^	[39]
3D-PP genosensor	Graphene	RNA	RNA COVID-19	-	0.015 μmol L^−1^	[36]
Gpt-PLA	Graphite	Antibody	Protein spike S1 COVID-19	Synthetic saliva	1.3 nmol L^−1^	[58]
3D genosensor	Graphene	cDNA	cDNA COVID-19	Synthetic saliva and synthetic saliva		[59]
3D-printed cell	CB	Anti-N Anti-S_RBD_ proteins S_RBD_	N S_RBD_ proteins anti-S_RBD_	Saliva	5 pg mL^−1^ 1 pg mL^−1^ 0.1 pg mL^−1^	[40]
3D printed biosensor	CB	Antibody	Protein spike S1	Human serum Synthetic saliva	2.7 μmol L^−1^	[38]
3DnCes	Nanocarbon	DNA	1-naphthol	-	-	[60]
3D-printed biodevice	CB	Anti-A29 DNA MKPV	Protein A29 DNA MKPV	Human serum	2.7 nmol L^−1^ 0.029 μmol L^−1^	[37]
3D PLA-GDMF-EC	Graphene	Anti-Park7/DJ-1	Park7/DJ-1 Parkinson’s disease	Blood serum and cerebrospinal fluid	1.01 µg L^−1^	[61]
3D r-PLA	CB and CNTs	cDNA	YFV cDNA	Human serum	0.138 μmol L^−1^	[43]

The abbreviations of the sensors correspond to the information provided by the authors. Additional information is available in the corresponding references. DMF-EC/AuNPs/HRP: chemically–electrochemically activated 3D-printed electrode modified with gold nanoparticles and horseradish peroxidase. G-PLA: 3D-printed graphene–polylactic acid electrode. Tyr-DHP/rGOPLA: reduced graphene oxide–polylactic acid 3D-printed electrode modified with DHP and tyrosinase. L-AAO@3D-nCEs: L-amino acid oxidase modified 3D-printed nanocomposite carbon electrode. Fe_3_O_4_@COF@BSA/3DE: magnetic COF and BSA-based 3D-printed electrode. SS electrode: skyscraper immunosensor. GOx/3DE: glucose oxidase-modified 3D-printed electrode. 3DnCes: 3D-printed nanocarbon electrodes. 3D PLA-GDMF-EC: chemically and electrochemically treated graphene–polylactic acid 3D-printed electrode. 3D r-PL: recycled PLA 3D printed electrode.

## References

[1] Madhurantakam S., Muthukumar S., Prasad S. (2022). Emerging Electrochemical Biosensing Trends for Rapid Diagnosis of COVID-19 Biomarkers as Point-of-Care Platforms: A Critical Review. ACS Omega.

[2] Tripathy S., Singh S.G. (2020). Label-Free Electrochemical Detection of DNA Hybridization: A Method for COVID-19 Diagnosis. Trans. Indian Natl. Acad. Eng..

[3] Brazaca L.C., dos Santos P.L., de Oliveira P.R., Rocha D.P., Stefano J.S., Kalinke C., Abarza Muñoz R.A., Bonacin J.A., Janegitz B.C., Carrilho E. (2021). Biosensing Strategies for the Electrochemical Detection of Viruses and Viral Diseases—A Review. Anal. Chim. Acta.

[4] Da Silva E.T.S.G., Souto D.E.P., Barragan J.T.C., de Fátima Giarola J., de Moraes A.C.M., Kubota L.T. (2017). Electrochemical Biosensors in Point-of-Care Devices: Recent Advances and Future Trends. ChemElectroChem.

[5] Burcu Bahadir E., Kemal Sezgintürk M. (2015). Applications of Electrochemical Immunosensors for Early Clinical Diagnostics. Talanta.

[6] Ribeiro B.V., Cordeiro T.A.R., Freitas G.R.O.E., Ferreira L.F., Franco D.L. (2020). Biosensors for the Detection of Respiratory Viruses: A Review. Talanta Open.

[7] Stefano J.S., Silva L.R.G.E., Kalinke C., de Oliveira P.R., Crapnell R.D., Brazaca L.C., Bonacin J.A., Campuzano S., Banks C.E., Janegitz B.C. (2023). Human Monkeypox Virus: Detection Methods and Perspectives for Diagnostics. TrAC Trends Anal. Chem..

[8] Yang Z., Zhang X., Guo J. (2022). Functionalized Carbon-Based Electrochemical Sensors for Food and Alcoholic Beverage Safety. Appl. Sci..

[9] Pumera M., Sánchez S., Ichinose I., Tang J. (2007). Electrochemical Nanobiosensors. Sensors Actuators B Chem..

[10] Bollella P., Fusco G., Tortolini C., Sanzò G., Favero G., Gorton L., Antiochia R. (2017). Beyond Graphene: Electrochemical Sensors and Biosensors for Biomarkers Detection. Biosens. Bioelectron..

[11] Kimmel D.W., Leblanc G., Meschievitz M.E., Cliffel D.E. (2012). Electrochemical Sensors and Biosensors. Anal. Chem..

[12] Stefano J.S., Orzari L.O., Silva-Neto H.A., de Ataíde V.N., Mendes L.F., Coltro W.K.T., Longo Cesar Paixão T.R., Janegitz B.C. (2022). Different Approaches for Fabrication of Low-Cost Electrochemical Sensors. Curr. Opin. Electrochem..

[13] Lahcen A.A., Rauf S., Beduk T., Durmus C., Aljedaibi A., Timur S., Alshareef H.N., Amine A., Wolfbeis O.S., Salama K.N. (2020). Electrochemical Sensors and Biosensors Using Laser-Derived Graphene: A Comprehensive Review. Biosens. Bioelectron..

[14] Wanjari V.P., Reddy A.S., Duttagupta S.P., Singh S.P. (2023). Laser-Induced Graphene-Based Electrochemical Biosensors for Environmental Applications: A Perspective. Environ. Sci. Pollut. Res..

[15] Komuro N., Takaki S., Suzuki K., Citterio D. (2013). Inkjet Printed (Bio)Chemical Sensing Devices. Anal. Bioanal. Chem..

[16] Tortorich R.P., Shamkhalichenar H., Choi J.W. (2018). Inkjet-Printed and Paper-Based Electrochemical Sensors. Appl. Sci..

[17] Hayat A., Marty J.L. (2014). Disposable Screen Printed Electrochemical Sensors: Tools for Environmental Monitoring. Sensors.

[18] Beitollahi H., Mohammadi S.Z., Safaei M., Tajik S. (2020). Applications of Electrochemical Sensors and Biosensors Based on Modified Screen-Printed Electrodes: A Review. Anal. Methods.

[19] Sinha A., Dhanjai, Stavrakis A.K., Stojanović G.M. (2022). Textile-Based Electrochemical Sensors and Their Applications. Talanta.

[20] Liu X., Yao Y., Ying Y., Ping J. (2019). Recent Advances in Nanomaterial-Enabled Screen-Printed Electrochemical Sensors for Heavy Metal Detection. TrAC Trends Anal. Chem..

[21] Cardoso R.M., Kalinke C., Rocha R.G., dos Santos P.L., Rocha D.P., Oliveira P.R., Janegitz B.C., Bonacin J.A., Richter E.M., Munoz R.A.A. (2020). Additive-Manufactured (3D-Printed) Electrochemical Sensors: A Critical Review. Anal. Chim. Acta.

[22] Stefano J.S., Kalinke C., Da Rocha R.G., Rocha D.P., Da Silva V.A.O.P., Bonacin J.A., Angnes L., Richter E.M., Janegitz B.C., Muñoz R.A.A. (2022). Electrochemical (Bio)Sensors Enabled by Fused Deposition Modeling-Based 3D Printing: A Guide to Selecting Designs, Printing Parameters, and Post-Treatment Protocols. Anal. Chem..

[23] Abdalla A., Patel B.A. (2020). 3D-Printed Electrochemical Sensors: A New Horizon for Measurement of Biomolecules. Curr. Opin. Electrochem..

[24] Rocha D.P., Rocha R.G., Castro S.V.F., Trindade M.A.G., Munoz R.A.A., Richter E.M., Angnes L. (2021). Posttreatment of 3D-printed Surfaces for Electrochemical Applications: A Critical Review on Proposed Protocols. Electrochem. Sci. Adv..

[25] Agarwal R. (2022). The Personal Protective Equipment Fabricated via 3D Printing Technology during COVID-19. Ann. 3D Print. Med..

[26] Pedraja J., Maestre J.M., Rabanal J.M., Morales C., Aparicio J., del Moral I. (2020). Role of 3D Printing in the Protection of Surgical and Critical Care Professionals in the COVID-19 Pandemic. Rev. Española Anestesiol. Reanim..

[27] Longhitano G.A., Nunes G.B., Candido G., da Silva J.V.L. (2021). The Role of 3D Printing during COVID-19 Pandemic: A Review. Prog. Addit. Manuf..

[28] Siddique S.H., Hazell P.J., Wang H., Escobedo J.P., Ameri A.A.H. (2022). Lessons from Nature: 3D Printed Bio-Inspired Porous Structures for Impact Energy Absorption—A Review. Addit. Manuf..

[29] Perales-Rondon J.V., Rojas D., Gao W., Pumera M. (2023). Copper 3D-Printed Electrodes for Ammonia Electrosynthesis via Nitrate Reduction. ACS Sustain. Chem. Eng..

[30] Kumar A., Padinjareveetil K., Perales-Rondon J.V., Pumera M., Padinjareveetil A.K.K., Perales-Rondon J.V., Pumera M. (2023). Engineering 3D Printed Structures Towards Electrochemically Driven Green Ammonia Synthesis: A Perspective. Adv. Mater. Technol..

[31] Zambiazi P.J., de Moraes A.T.N., Kogachi R.R., Aparecido G.O., Formiga A.L.B., Bonacin J.A. (2020). Performance of Water Oxidation by 3D Printed Electrodes Modified by Prussian Blue Analogues. J. Braz. Chem. Soc..

[32] Hughes J.P., Dos Santos P.L., Down M.P., Foster C.W., Bonacin J.A., Keefe E.M., Rowley-Neale S.J., Banks C.E. (2019). Single Step Additive Manufacturing (3D Printing) of Electrocatalytic Anodes and Cathodes for Efficient Water Splitting. Sustain. Energy Fuels.

[33] Tian X., Jin J., Yuan S., Chua C.K., Tor S.B., Zhou K. (2017). Emerging 3D-Printed Electrochemical Energy Storage Devices: A Critical Review. Adv. Energy Mater..

[34] Mazzanti V., Malagutti L., Mollica F. (2019). FDM 3D Printing of Polymers Containing Natural Fillers: A Review of Their Mechanical Properties. Polymers.

[35] Ambrosi A., Pumera M. (2016). 3D-Printing Technologies for Electrochemical Applications. Chem. Soc. Rev..

[36] Crevillen A.G., Mayorga-Martinez C.C., Vaghasiya J.V., Pumera M. (2022). 3D-Printed SARS-CoV-2 RNA Genosensing Microfluidic System. Adv. Mater. Technol..

[37] Silva L.R.G., Stefano J.S., Kalinke C., Crapnell R.D., Brazaca L.C., Marcolino-Junior L.H., Bergamini M.F., Banks C.E., Janegitz B.C. (2024). Dual-Target Additively Manufactured Electrochemical Sensor for the Multiplexed Detection of Protein A29 and DNA of Human Monkeypox Virus. ACS Omega.

[38] Silva L.R.G., Stefano J.S., Crapnell R.D., Banks C.E., Janegitz B.C. (2023). Additive Manufacturing of Carbon Black Immunosensors Based on Covalent Immobilization for Portable Electrochemical Detection of SARS-CoV-2 Spike S1 Protein. Talanta Open.

[39] Muñoz J., Pumera M. (2021). 3D-Printed COVID-19 Immunosensors with Electronic Readout. Chem. Eng. J..

[40] De Matos Morawski F., Martins G., Ramos M.K., Zarbin A.J.G., Blanes L., Bergamini M.F., Marcolino-Junior L.H. (2023). A Versatile 3D Printed Multi-Electrode Cell for Determination of Three COVID-19 Biomarkers. Anal. Chim. Acta.

[41] Crapnell R.D., Sigley E., Williams R.J., Brine T., Garcia-Miranda Ferrari A., Kalinke C., Janegitz B.C., Bonacin J.A., Banks C.E. (2023). Circular Economy Electrochemistry: Recycling Old Mixed Material Additively Manufactured Sensors into New Electroanalytical Sensing Platforms. ACS Sustain. Chem. Eng..

[42] Sigley E., Kalinke C., Crapnell R.D., Whittingham M.J., Williams R.J., Keefe E.M., Janegitz B.C., Bonacin J.A., Banks C.E. (2023). Circular Economy Electrochemistry: Creating Additive Manufacturing Feedstocks for Caffeine Detection from Post-Industrial Coffee Pod Waste. ACS Sustain. Chem. Eng..

[43] Kalinke C., Crapnell R.D., Sigley E., Whittingham M.J., de Oliveira P.R., Brazaca L.C., Janegitz B.C., Bonacin J.A., Banks C.E. (2023). Recycled Additive Manufacturing Feedstocks with Carboxylated Multi-Walled Carbon Nanotubes toward the Detection of Yellow Fever Virus CDNA. Chem. Eng. J..

[44] Crapnell R.D., Kalinke C., Silva L.R.G., Stefano J.S., Williams R.J., Abarza Munoz R.A., Bonacin J.A., Janegitz B.C., Banks C.E. (2023). Additive Manufacturing Electrochemistry: An Overview of Producing Bespoke Conductive Additive Manufacturing Filaments. Mater. Today.

[45] Katseli V., Economou A., Kokkinos C. (2019). Single-Step Fabrication of an Integrated 3D-Printed Device for Electrochemical Sensing Applications. Electrochem. Commun..

[46] Manzanares-Palenzuela C.L., Hermanova S., Sofer Z., Pumera M. (2019). Proteinase-Sculptured 3D-Printed Graphene/Polylactic Acid Electrodes as Potential Biosensing Platforms: Towards Enzymatic Modeling of 3D-Printed Structures. Nanoscale.

[47] López Marzo A.M., Mayorga-Martinez C.C., Pumera M. (2020). 3D-Printed Graphene Direct Electron Transfer Enzyme Biosensors. Biosens. Bioelectron..

[48] Cardoso R.M., Silva P.R.L., Lima A.P., Rocha D.P., Oliveira T.C., do Prado T.M., Fava E.L., Fatibello-Filho O., Richter E.M., Muñoz R.A.A. (2020). 3D-Printed Graphene/Polylactic Acid Electrode for Bioanalysis: Biosensing of Glucose and Simultaneous Determination of Uric Acid and Nitrite in Biological Fluids. Sens. Actuators B Chem..

[49] Silva V.A.O.P., Fernandes-Junior W.S., Rocha D.P., Stefano J.S., Munoz R.A.A., Bonacin J.A., Janegitz B.C. (2020). 3D-Printed Reduced Graphene Oxide/Polylactic Acid Electrodes: A New Prototyped Platform for Sensing and Biosensing Applications. Biosens. Bioelectron..

[50] Koukouviti E., Kokkinos C. (2021). 3D Printed Enzymatic Microchip for Multiplexed Electrochemical Biosensing. Anal. Chim. Acta.

[51] Muñoz J., Redondo E., Pumera M. (2021). Chiral 3D-Printed Bioelectrodes. Adv. Funct. Mater..

[52] Wang L., Pumera M. (2021). Covalently Modified Enzymatic 3D-Printed Bioelectrode. Microchim. Acta.

[53] Wang L., Gao W., Ng S., Pumera M. (2021). Chiral Protein-Covalent Organic Framework 3D-Printed Structures as Chiral Biosensors. Anal. Chem..

[54] Domingo-Roca R., Macdonald A.R., Hannah S., Corrigan D.K. (2022). Integrated Multi-Material Portable 3D-Printed Platform for Electrochemical Detection of Dopamine and Glucose. Analyst.

[55] Calabria D., Lazzarini E., Pace A., Trozzi I., Zangheri M., Cinti S., Difonzo M., Valenti G., Guardigli M., Paolucci F. (2023). Smartphone-Based 3D-Printed Electrochemiluminescence Enzyme Biosensor for Reagentless Glucose Quantification in Real Matrices. Biosens. Bioelectron..

[56] Hussain K.K., Hopkins R., Yeoman M.S., Patel B.A. (2024). 3D Printed Skyscraper Electrochemical Biosensor for the Detection of Tumour Necrosis Factor Alpha (TNFα) in Faeces. Sens. Actuators B Chem..

[57] Martins G., Gogola J.L., Budni L.H., Janegitz B.C., Marcolino-Junior L.H., Bergamini M.F. (2021). 3D-Printed Electrode as a New Platform for Electrochemical Immunosensors for Virus Detection. Anal. Chim. Acta.

[58] Stefano J.S., Guterres e Silva L.R., Rocha R.G., Brazaca L.C., Richter E.M., Abarza Muñoz R.A., Janegitz B.C. (2021). New Conductive Filament Ready-to-Use for 3D-Printing Electrochemical (Bio)Sensors: Towards the Detection of SARS-CoV-2. Anal. Chim. Acta.

[59] Silva L.R.G., Stefano J.S., Orzari L.O., Brazaca L.C., Carrilho E., Marcolino-Junior L.H., Bergamini M.F., Munoz R.A.A., Janegitz B.C. (2022). Electrochemical Biosensor for SARS-CoV-2 CDNA Detection Using AuPs-Modified 3D-Printed Graphene Electrodes. Biosensors.

[60] Jyoti, Fojta M., Hermanová M., Pivoňková H., Alduhaish O., Pumera M. (2023). Genosensing on a 3D-Printed Nanocarbon Electrode. Electrochem. Commun..

[61] Kalinke C., De Oliveira P.R., Banks C.E., Janegitz B.C., Bonacin J.A. (2023). 3D-Printed Immunosensor for the Diagnosis of Parkinson’s Disease. Sens. Actuators B Chem..

[62] Kalinke C., de Oliveira P.R., Neumsteir N.V., Henriques B.F., de Oliveira Aparecido G., Loureiro H.C., Janegitz B.C., Bonacin J.A. (2022). Influence of Filament Aging and Conductive Additive in 3D Printed Sensors. Anal. Chim. Acta.

